# The impact of depression and ghrelin on body weight in migraineurs

**DOI:** 10.1186/1129-2377-15-23

**Published:** 2014-04-24

**Authors:** Bulent Turan, Zeynep Osar Siva, Derya Uluduz, Dildar Konukoglu, Feyza Erenler, Sabahattin Saip, Baki Goksan, Aksel Siva

**Affiliations:** 1 Department of Psychology, University of Alabama at Birmingham, Birmingham, AL, USA; 2 Internal Medicine Department, Istanbul University Cerrahpaşa School of Medicine, Istanbul, Turkey; 3 Neurology Department, Istanbul University Cerrahpaşa School of Medicine, Istanbul, Turkey; 4 Biochemistry Department, Istanbul University Cerrahpaşa School of Medicine, Istanbul, Turkey; 5 Undergraduate Medical Student, Istanbul University Cerrahpaşa School of Medicine, Istanbul, Turkey

**Keywords:** Body weight, Ghrelin, Migraine, Psychiatric comorbidity, Depression, Anxiety

## Abstract

**Background:**

Comorbidity of migraine with anxiety and depression may play a role in the link between migraine and obesity. We examined the moderating and mediating roles of ghrelin in the relationship between depression (and anxiety) and body weight in newly diagnosed migraineurs.

**Methods:**

Participants were 63 newly diagnosed migraine patients (using the ICHD-II criteria) and 42 healthy volunteers. Body mass index (BMI) was calculated by measuring height and weight. Ghrelin was assessed at fasting. Depression was assessed with the Hamilton Depression scale, and anxiety with the Hamilton Anxiety scale.

**Results:**

The data did not support the mediating role of ghrelin in the relationship between depression (or anxiety) and BMI for either the migraine or the control group. The interaction between ghrelin and depression as well as anxiety was significant for the migraine group, but not for the control group. Depressed (or anxious) migraineurs had a positive association between ghrelin and BMI, whereas for the non-depressed (or non-anxious) migraineurs this association was negative.

**Conclusions:**

Depression and anxiety moderated the effect of ghrelin on BMI for migraineurs. Management of anxiety and depression might be regarded as part of migraine treatment.

## Background

Migraine is a recurrent neurovascular disorder with moderate to severe throbbing and mostly unilateral headache with or without photo-phonophobia and nausea [1]. The pathophysiology of migraine is not fully understood. However, it has long been known that migraine attacks may be precipitated by different triggers, such as physical and psychological stressors [2-4]. A number of studies reported increased prevalence of migraine in individuals with depression and anxiety [5-10]. Comorbidity of migraine with metabolic disorders, such as obesity and insulin resistance, has also been reported [11]. In patients suffering from migraine, increase in body weight is associated with higher headache frequency and severity, and with higher levels of disability [12]. A recent study by Peterlin et al. suggested that episodic migraine rates are increased with obesity [13]. Population based studies also suggest that obesity may be a risk for the progression of episodic migraine to chronic migraine [14,15].

The neurobiological mechanisms underlying the relationship between migraine and obesity have not been elucidated precisely. However, the known increased comorbidity of anxiety and depression with migraine may be a link between migraine and altered feeding behavior leading to obesity. It has been reported that there is a close relationship between emotional disorders and increased visceral fat accumulation [16]. Anxiety and depression associated with migraine may contribute to increased food intake in migraineurs by affecting appetite regulators. The orexigenic gut hormone ghrelin is an important modulator of energy intake [17]. It has been reported that circulating ghrelin levels increase not only in response to energy insufficiency but also in response to acute and chronic stress. Moreover, increasing ghrelin levels produce anxiolytic and antidepressant-like responses. Chronic stress causes elevated ghrelin levels, and the effects of depression and anxiety are minimized when ghrelin levels rise [18].

In this study, we aimed to examine the role of chronic anxiety and depression on body weight, and the moderating and mediating roles of ghrelin in these relationships in newly diagnosed, otherwise healthy, non-diabetic migraine patients. It is possible that ghrelin mediates the effect of depression and anxiety on body weight in migraineurs. However, another possibility that has received little attention in the literature is that ghrelin moderates the effect of anxiety and depression on body weight in migraineurs. In other words, anxiety and depression may have a statistical interaction with ghrelin such that the effect of ghrelin on body weight is stronger for depressed (or anxious) migraine patients compared to non-depressed (or non-anxious) migraine patients, and depressed (or anxious) migraine patients who also have high levels of ghrelin may be more vulnerable to obesity.

## Methods

Three hundred seventy four consecutive migraine patients attending for the first time the tertiary Headache Center of Istanbul University Neurology Department (Turkey) from January 2010 to January 2011 were screened for the study. Migraine was diagnosed according to the ICHD-II criteria [19]. Participants were excluded if they were: (1) diabetic [20], had any endocrinological disease that might interfere with insulin resistance, (2) had a history of major psychiatric disorders, chronic renal or hepatic disease, malignancy, acute cardiovascular events, or a neurological disease; (4) were using any medication that might interfere with insulin resistance; or (5) were using prophylactic antimigraine treatment. BMI is one of the medical standards for measuring adiposity and defining obesity. Body weight (kg) and height (cm) was measured during the study visit by a research assistant, and BMI was calculated by dividing weight by height squared [21]. From the 374 patients screened, 63 eligible patients (7 men, 56 women; mean age = 33.94 ± 8.85 years) participated. The patients underwent extensive face to face physical, neurological, and psychological examinations by an expert endocrinologist, a neurologist specialized in headache disorders, and a clinical psychologist. According to the duration and frequency of days with headache per month, headache was further classified as episodic or chronic migraine for supplemental analyses.

Our main analyses were on the data from this sample of migraine patients. We also had a control group, which consisted of 42 healthy volunteers (9 men, 33 women; mean age = 32.31 ± 8.91 yrs). All participants were Turkish/Caucasian. There were no significant differences between the groups in smoking status or in the number of diabetes risk factors [20]. The study was approved by the local Ethics Committee of Istanbul University Cerrahpasa School of Medicine and informed consent was obtained from participants.

For supplementary analyses, the migraine patients were further categorized as episodic versus chronic migraine based on the definition in the ICHD-II criteria [19]. Episodic and chronic migraine patients were not significantly different in any of the study variables (see Tables 1 and 2). Fasting plasma ghrelin concentrations were determined by Enzyme Immunoassay according to the manufacturer’s guidelines (*Bachem, Peninsula Laboratories, and LLC, USA*). The results are reported as pg/mL and minimum detectable levels were 0.45 pg/mL. The mean CV was less than 7.5%.

**Table 1:** Baseline characteristics of the migraine and control groups

	**Control group (n = 42)**	**Migraine group (n = 63)**	**p value**	**Episodic migraine only (n = 43)**	**Chronic migraine only (n = 20)**	**p value**
Age (years)	32.31 ± 8.9	33.94 ± 8.8	0.36	33.21 ± 8.7	35.50 ± 9.2	0.34
Gender (male/female)	9/34	7/56	0.17	3/40	4/16	0.20
BMI	23.22 ± 3.48	25.48 ± 4.55	0.01	25.15 ± 4.39	26.18 ± 4.90	0.41
Waist circumference	78.85 ± 9.47	83.47 ± 11.31	0.04	82.35 ± 11.26	85.94 ± 11.34	0.27
Female	77.79 ± 9.12	82.20 ± 11.13		81.97 ± 11.45	82.79 ± 10.61	
Male	83.86 ± 10.21	92.71 ± 8.32		87.00 ± 8.66	97.00 ± 5.60	

**Table 2:** Ghrelin levels, Hamilton Depression scale (HAM-D) scores, and Hamilton Anxiety scale (HAM-A) scores for the study groups

	**Control group (n = 42)**	**Migraine group (n = 63)**	**p value**	**Episodic migraine only (n = 43)**	**Chronic migraine only (n = 20)**	**p value**
Ghrelin fasting (pgmol/l)	171.81 ± 95.1	187.31 ± 78.8	0.46	189.23 ± 77.9	183.18 ± 82.7	0.78
HAM-D	4.31 ± 4.2	11.90 ± 7.0	<0.001	11.46 ± 6.4	11.69 ± 6.8	0.68
HAM-A	5.45 ± 7.5	15.79 ± 8.8	<0.001	16.21 ± 8.5	15.66 ± 8.1	0.72

Depression was assessed with the Hamilton Depression (HAM-D) scale and anxiety was assessed with the Hamilton Anxiety (HAM-A) scale [22,23]. HAM-D scale is a 17 item- questionnaire, and was administered by a neurologist during a face to face interview. The minimum score is 0 and the maximum score is 53 points. The HAM-A probes 14 parameters and each item is scored on a 5-point scale, ranging from 0 = not present to 4 = severe. The scores from all 14 parameters are summed. The recommended cut-off score of 15 was used to create two groups: (a) without anxiety, and (b) with moderate to severe anxiety. Similarly, a cut-off score of 13 was used for HAM-D to create two groups: (a) not depressed, and (b) moderately to very severely depressed. Results were similar when continuous scores were used instead of these categories in the analyses.

Statistical analyses were conducted using the SPSS software (version 20). All analyses were two-tailed with α = 0.05. Mediation analyses were used to test the hypothesis that ghrelin mediates the effect of depression (or of anxiety) on BMI in migraineurs (and separately for the control group for comparison purposes). A mediation requires meeting all of the following four conditions: (a) The predictor (in this case depression or anxiety) is associated with the outcome (in this case BMI), (b) the predictor is associated with the mediator (in this case ghrelin), (c) the mediator is associated with the outcome while controlling for the predictor, and (d) the effect of the predictor on the outcome becomes non-significant (or significantly smaller) when the mediator is used as a control variable. These conditions were tested using multiple regression analyses.

Moderation analyses were conducted to test for a statistical interaction between depression (or anxiety) and ghrelin in predicting BMI [24]. This was done using multiple regression analyses—separately for the migraine and control groups—with BMI as the dependent variable. The independent variables were ghrelin at fasting, depression status (depressed versus not depressed), and the interaction of these two independent variables. Similar analyses were also conducted using the dichotomized anxiety variable instead of depression. Ghrelin scores were centered around their mean. Age and gender were also entered as control variables. Whenever the interaction term was significant, follow-up simple slope analyses were conducted to examine the relationship between ghrelin and BMI at high and low levels of depression (or anxiety).

## Results

The baseline characteristics of the migraine and control groups are presented in Table 1. There were no significant differences between the migraine and control groups in terms of age and gender (t = .92, p = .36, and *Χ*^2^ = 2.01, p = .17, respectively). Nevertheless, age and gender were controlled in all analyses, since most of our analyses were done separately for the migraine and control groups. Table 2 presents mean HAM-A and HAM-D scores, and ghrelin levels. HAM-A and HAM-D scores were significantly higher in migraineurs compared to controls (p < .001).

Only two of the 42 control group participants were in the depressed category (i.e., moderately to severely depressed), whereas 31 of the 63 migraine patients were moderately to severely depressed. Similarly, only three of the 42 control group participants had moderate to severe anxiety compared to 24 of the 63 migraine patients. Neither the two depression groups nor the two anxiety groups had significantly different BMI values for the migraine group (F = 2.64, p = .11, and F = .66, p = .42, respectively, controlling for gender and age). The control group also did not show differences in BMI according to depression or anxiety status (F = 1.36, p = .25, and F = .04, p = .84, respectively, controlling for gender and age). Thus, the first condition for mediation was not satisfied, and therefore no claims to the mediating role of ghrelin on the effect of depression or anxiety on BMI could be made. Further analyses revealed that the data did not satisfy the third and fourth conditions of mediation either. Furthermore, using newer, more powerful bootstrapping techniques also did not reveal significant indirect effects of depression or anxiety on BMI through ghrelin. Thus, the data did not support the mediating role of ghrelin in the relationship between depression (or anxiety) and BMI for either the migraine or the control group.

We then tested the hypothesis that depression (or anxiety) moderates the effect of ghrelin on BMI for migraine patients. A moderation hypothesis means that there is a statistical interaction between depression and ghrelin in predicting BMI [24]. In other words, the effect of ghrelin on BMI is different for depressed and non-depressed participants. A regression analysis was conducted—separately for the migraine and control groups—with BMI serving as the outcome variable. The independent variables were ghrelin at fasting (centered), depression status, and the interaction of these two independent variables. Age and gender were also entered as control variables. This analysis revealed a significant interaction term (B = .04, *t* = 2.50, *p* = .02) for the migraine group, whereas for the control group, the interaction was not significant (B = .01, *t* = .18, *p* = .84). To further probe this significant interaction for the migraine group, follow-up simple slope analyses were conducted for the depressed and non-depressed migraine patients.

**Figure 1:**
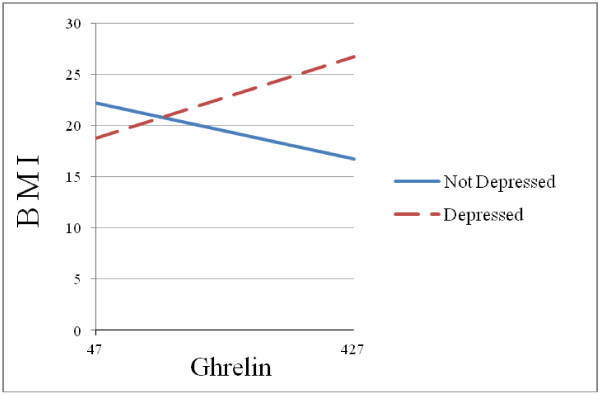
Depression status moderates the effect of ghrelin on BMI.

These analyses revealed that for depressed migraineurs ghrelin had a positive association with BMI (B = .02, *t* = 1.82, *p* = .07), whereas for non-depressed migraineurs the association between ghrelin and BMI was negative (B = -.01, *t* = -1.76, *p* = .08). Figure 1 depicts this significant interaction for the migraine group.

**Figure 2:**
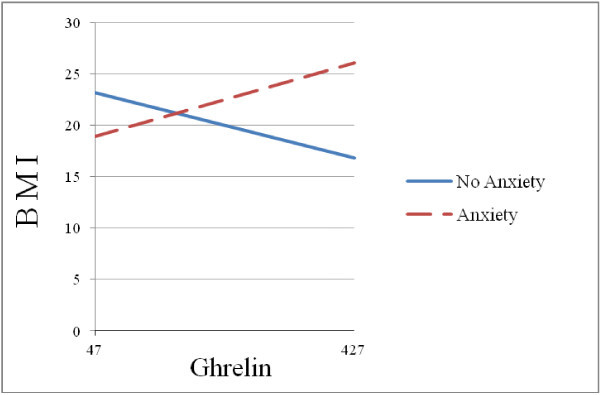
Anxiety status moderates the effect of ghrelin on BMI.

Similar analyses using anxiety instead of depression yielded parallel results (Figure 2). The interaction between anxiety status and baseline ghrelin was significant for the migraine group (B = .04, *t* = 2.59, *p* = .01), but not for the control group, (B = .00 *t* = -.05, *p* = .96). Follow-up simple slope analyses for the migraine group revealed that for migraineurs with anxiety ghrelin had a positive association with BMI (B = .02, *t* = 1.83, *p* = .07), whereas for migraineurs without anxiety the association between ghrelin and BMI was negative (B = -.02, *t* = -1.86, *p* = .07).

## Discussion

Migraine and obesity represent two major public health problems. Moreover, both chronic migraine and obesity are associated with increased risk of comorbidities and have a significant impact on the health care system [25].

There is an established relationship between obesity and headache. Data on the prevalence of migraine in obese patients are inconclusive. However, it has been shown that obesity is associated with an increased frequency and intensity of migraine attacks [26]. It has been reported that morbidly obese women have a high incidence of migraine, especially migraine with aura [27]. The same association was found in overweight adolescent females who showed an almost fourfold risk for migraine when compared with normal-weight girls [28]. In a large population-based study focusing on the relationship between BMI and episodic migraine, a total of 30,215 participants were interviewed, and 3,791 were diagnosed with migraine. BMI was not associated with the prevalence of episodic migraine. However, it was associated with the frequency of headache attacks, higher chances of aura, and severe pain [12]. In another large study with a sample of 63,467 women (ages 45 years or older, of which,14.5% had migraine), migraine prevalence did not differ significantly as a function of BMI, but severely obese women were more likely to have increased headache frequency and risk of phonophobia and photophobia [29]. Moreover, obesity is regarded as a major risk factor for chronification of episodic migraine in adults and children [26].

Pathophysiological mechanisms that might be responsible for the association between migraine and obesity are not fully established yet. It has been reported that certain psychological conditions can play a role in the migraine-obesity relationship. There is emerging evidence for a possible association between stress and increased risk of both headache and obesity. Studies have shown that obesity and depression, depression and chronic daily headache, and chronic daily headache and obesity are linked [26]. Indeed, in a carefully conducted study, the relationship of obesity with migraine frequency and migraine-related disability was modified by depression and anxiety, with the strongest effect observed in migraineurs with both depression and anxiety. It was concluded that the psychological comorbidities are a common problem in migraine patients and have an additive effect on the risk of migraine progression [26,30]. The prevalence of major depression has been reported in a number of studies to be 1.7 fold or higher among individuals with migraine compared to non-migraineurs [8]. In a recent study Ligthart and associates analyzed a cohort with different types of chronic pain and concluded that anxiety and depression can explain a considerable part of the comorbidity of migraine and other types of pain [6,7,31].

In the present study depression and anxiety assessed with HAM-D and HAM-A were significantly higher in migraine patients compared to controls. We hypothesized that the psychological comorbidities in migraineurs may be a factor leading to increased energy intake and future obesity. In a previous study it has been reported that there is a close relationship between increased visceral fat accumulation and emotional disorders [16]. It has also been shown that in migraineurs, there is a tendency for increased appetite [32]. Anxiety and depression in migraine may facilitate weight gain through their interaction with central mechanisms inducing appetite. Central regulators of energy intake may be a link between migraine and increased energy intake. We found that depression and anxiety were not associated with BMI in our sample.

We tested the hypothesis that depression and anxiety moderate the effect of ghrelin on BMI for non-diabetic migraine patients and found support for this hypothesis. Our results revealed a statistically significant interaction between depression (as well as with anxiety) and ghrelin in predicting BMI for the migraine group, but not for the control group. That is, the effect of ghrelin on BMI was different for depressed and non-depressed migraineurs. For depressed migraineurs ghrelin had a positive association with BMI, whereas for non-depressed migraineurs the association between ghrelin and BMI was negative. Moreover, post-hoc exploratory analyses suggested that the chronic migraine group is mainly responsible for the interaction effect found. A similar interaction effect was found for anxiety as well.

The brain-gut axis regulates appetite and maintains energy homeostasis through communication between the gut and the central nervous system. Dysregulation of the brain-gut axis is associated with metabolic imbalances found in eating disorders as well as in association with stress, anxiety and depression [33]. The orexigenic gut hormone ghrelin is an important central modulator of energy intake [17]. Ghrelin is expressed mainly in the arcuate nucleus of the hypothalamus although ghrelin positive neurons are also found in the lateral hypothalamus and paraventricular nucleus. Fasting causes ghrelin to be released from the gastrointestinal tract, and the hormone then plays a role in sending hunger signals to the brain. It has been reported that circulating ghrelin levels increase not only in response to energy insufficiency but also in response to acute and chronic stress [34]. The role of ghrelin in depression and anxiety has been reported by a number of studies. In experimental studies, augmented ghrelin levels were found in mice following chronic defeat stress, and it was suggested that ghrelin might be involved in the defense against stress induced depression and anxiety. Ghrelin infusion into the lateral ventricle produced an anxiolytic like effect and inhibition of ghrelin induced an increase in depression and anxiety like behaviors in rats.

Human studies on ghrelin and stress suggest the coordinating role of ghrelin on behavioral response to stress by modulating energy intake. In one study, patients suffering from major depression had lower plasma ghrelin levels and antidepressant effects were reported following ghrelin administration [35]. Chronic stress causes ghrelin levels to go up and behaviors associated with depression and anxiety decrease when ghrelin levels rise. An unfortunate side effect, however, is increased food intake and body weight [36]. In mice, the stress-induced rises in ghrelin lead to overeating and increased body weight, suggesting a mechanism for the increased prevalence of weight-related issues observed in humans with chronic stress and depression [18].

The diurnal pattern of ghrelin secretion plays an important role in meal initiation. In a previous study, Cummings and colleagues have sought to determine whether a single, conveniently obtainable plasma ghrelin value could serve as a surrogate for the integrated 24-h AUC ghrelin value in human subjects [36]. They reported that ghrelin levels rose nearly two-fold shortly before each meal and fall to trough levels within 1 h after eating, a diurnal profile that is consistent with a physiological role for ghrelin in initiating individual meals. They concluded that overnight fasting ghrelin levels measured early in the morning and 1st hour after breakfast both have a very high correlation with 24-h AUC values of the hormone (r = 0.873, P = 0.0004). We measured overnight fasting ghrelin levels. Therefore, we cannot make any conclusions about postprandial changes in ghrelin levels. However, we can assume that fasting ghrelin levels reflected the diurnal ghrelin dynamics.

Our findings did not support the mediating role of ghrelin in the relationship between anxiety (or depression) and BMI for either the migraine or the control group. However, our data revealed a significant interaction between ghrelin and anxiety and depression, such that migraine patients with depression (or anxiety) as well as high levels of ghrelin had higher BMI.

One limitation of this study is that ghrelin levels were assessed only in the beginning of headache treatment. Changes in ghrelin could also be examined after headache and/or depression treatment. In our control group, only two participants had depression and only three had anxiety. It should be noted, however, results were very similar, and conclusions the same, when we used continuous scores of symptom severity for depression and anxiety instead of using binary variables for depression and anxiety status. Furthermore, the important contribution of our data is in highlighting the interactive effect of ghrelin and depression (or anxiety) on BMI for the migraine group, which included a substantial number of patients with depression and anxiety.

The groups did not differ statistically in terms of gender distribution. Nevertheless, we statistically controlled for the effects of gender, since our sample size was not large. The headache and control groups differed in BMI, and we do not know whether or not this affected the results in comparing the headache group with the control group. However, as mentioned earlier, the important contribution of our data is in highlighting the interactive effect of ghrelin and depression (or anxiety) on BMI for the migraine group.

## Conclusion

Obesity should be kept under control as it is a cause of cardiovascular risk in migraine patients. Migraineurs should be motivated to maintain a normal weight for height and age or decrease it if overweight or obese. Reaching this goal will require sticking to sensible diets and receiving behavioral support. The management of anxiety and depression associated with migraine should be regarded as a part of migraine treatment. The use of migraine preventive medications that are weight-neutral or that are associated with weight loss in those who are overweight or obese may be recommended.

## Abbreviations

BMI: Body mass index; HAM-D: Hamilton Depression scale; HAM-A: Hamilton Anxiety scale.

## Competing interests

The authors declare that they have no competing interests.

## Authors’ contributions

BT: Supervised the statistical analyses and interpreted the data; contributed to writing and revising the manuscript. ZOS, DU: Involved in conception and design of the study, acquisition of the data, and writing the manuscript. DK: Organized the laboratory investigations, contributed to writing the laboratory Methods and Results sections. FE: Involved in organizing the patients’ appointments, performed BMI calculations. SS, BG, AS: Were involved in revising the manuscript for important intellectual content, contributed to study conception and design, and acquisition of data. All authors read and approved the final manuscript.
